# Rhythm Facilitates the Detection of Repeating Sound Patterns

**DOI:** 10.3389/fnins.2016.00009

**Published:** 2016-01-29

**Authors:** Vani G. Rajendran, Nicol S. Harper, Khaled H. A. Abdel-Latif, Jan W. H. Schnupp

**Affiliations:** Auditory Neuroscience Group, Department of Physiology, Anatomy, and Genetics, University of OxfordOxford, UK

**Keywords:** rhythm, pattern detection, temporal regularity, noise learning, psychoacoustics, modulation filters, footsteps, auditory neuroscience models

## Abstract

This study investigates the influence of temporal regularity on human listeners' ability to detect a repeating noise pattern embedded in statistically identical non-repeating noise. Human listeners were presented with white noise stimuli that either contained a frozen segment of noise that repeated in a temporally regular or irregular manner, or did not contain any repetition at all. Subjects were instructed to respond as soon as they detected any repetition in the stimulus. Pattern detection performance was best when repeated targets occurred in a temporally regular manner, suggesting that temporal regularity plays a facilitative role in pattern detection. A modulation filterbank model could account for these results.

## Introduction

Beneficial to survival in a complex and ever-changing acoustic environment is the ability to quickly identify relevant sounds that comprise the scene. One useful strategy is to detect recurring patterns over time, as these are often salient and suggestive of animate sound sources. Consider footsteps: steps on gravel sound nothing like steps through grass or through puddles, yet all of these very disparate sounds are easily recognized as the sound of footsteps if they occur in a rhythmic, repeating pattern. To recognize rhythmic patterns, the brain needs to search for recurrences of arbitrary and potentially complex sounds over timescales ranging from fractions of a second to tens of seconds.

Studies of auditory pattern detection often employ Gaussian white noise stimuli because they are spectrally broadband and devoid of prior meaning to listeners. Humans exhibit an impressive capacity to rapidly form recognition memories of frozen white noise tokens (Kaernbach, 2004; Agus and Pressnitzer, 2013), and these memories can persist for weeks (Agus et al., 2010). While human sensitivity to arbitrary repeating patterns has been well documented (Kaernbach, 2004; Chait et al., 2007; Agus et al., 2010; Agus and Pressnitzer, 2013), the question of how repetition is detected in the first place remains poorly understood. Previous noise learning studies have only explored conditions where repeating noise tokens were presented at precisely regular (isochronous) time intervals, and it is unclear whether such regularity is necessary or helpful for pattern detection. If sensory memory alone is responsible for pattern detection, then whether the sounds occur at regular or irregular intervals should have no effect on pattern detection.

However, the experiments described here reveal that detection performance does decline with increasing temporal irregularity, indicating that a sensitivity to slow temporal modulations or entrainment to the rhythmic structure of incoming sounds might facilitate pattern detection.

## Methods

The experimental methodology was approved by the local Ethical Review Committee of the Experimental Psychology Department of the University of Oxford, and conforms to the ethical standards in the 1964 Convention of Helsinki.

In order to investigate to what extent temporal regularity might facilitate pattern detection, we asked human subjects to detect repeating noise patterns played over headphones. We generated frozen noise “targets” and manipulated their regularity by embedding them in non-frozen “filler” noise of varying length. In this manner, we probed pattern detection in a temporally regular (REP-R) and temporally irregular or jittered (REP-J) context. REP-R stimuli were designed to measure how the detectability of a target depended on its duration relative to a fixed inter-onset interval (IOI). REP-J stimuli were designed to measure how the detectability of a target depended on the variability of IOI. Background “false alarm” detection rates were measured with a control stimulus of totally non-repeating noise (RAND). As a further control to test the subjects' ability to report changes in the quality of the noise stimuli rapidly and reliably, we also incorporated a fourth stimulus type in the experiment (PINK), in which the spectrum of the noise changed from white (flat amplitude spectrum) to pink (1/f amplitude spectrum). MATLAB was used for stimulus generation, response collection and data analysis.

**Figure 1:**
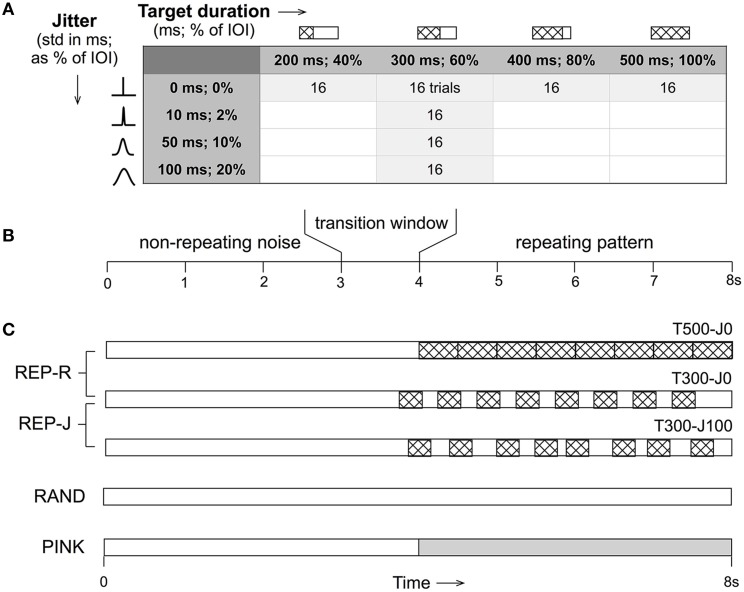
**(A)** For each of the parameter combinations shown for the REP conditions, 16 trials were generated. Target duration is displayed in milliseconds and as a percentage of the fixed inter-onset interval (IOI) of 500 ms. Jitter is shown as the standard deviation of the Gaussian distribution from which IOI durations are drawn in milliseconds, and as a percentage of the mean IOI of 500 ms. **(B)** All stimuli were 8 s in duration and began as non-repeating noise that then transitioned (if at all) at a random time between 3 and 4 s to a repeating section (or pink noise in the case of PINK). **(C)** Schematic representations of the different stimulus conditions where non-repeating noise is shown in white, repeating noise targets are patterned, and pink noise is shown in gray. T500-J0 (target duration of 500 ms, no jitter, top) is closest to the type of stimuli used in previous noise learning studies. A target duration of 300 ms (T300) was used in the jittered (REP-J) context. Control conditions were RAND (non-repeating white noise) and PINK (non-repeating white noise that transitions to pink noise). *N* = 16 trials for PINK and each of the 7 REP conditions, and *N* = 64 trials for RAND.

All stimuli were 8 s in duration, and either remained non-repeating noise throughout (RAND condition), or started with non-repeating noise for a variable (uniformly distributed over 3–4 s) duration before transitioning to alternating noise targets and fillers (REP conditions) or to pink noise (PINK condition). The repeating section contained exactly 8 repeats of a single noise target embedded in noise fillers with the duration and jitter parameter combinations shown in Figure 1. REP-R stimuli had target durations *T* of either 500, 400, 300, or 200 ms, and filler durations *F* = 500*-T* ms to yield a constant IOI of 500 ms. For the REP-J stimuli, *T* was fixed at 300 ms, and *F* was drawn independently from a Gaussian distribution with a mean of 200 ms and a standard deviation *J* of either 10, 50, or 100 ms. Thus, REP-J stimuli had normally-distributed random IOIs with a mean of 500 ms, while REP-R had a fixed IOI of 500 ms, corresponding to a repetition rate of ~2 Hz for all REP stimuli. Expressed as percentages, REP-R explored the detection of targets that were 100, 80, 60, or 40% of the IOI, and REP-J probed temporal jitter levels of 2, 10, or 20%, when quantified as the standard deviation of the IOI as a fraction of its mean. Examples of these stimuli can be found online^1^.

Subjects first underwent an instructional period during which the task was explained and examples of each stimulus type were played until the subjects reported that they could hear the repeating pattern on at least one occasion in both the REP-R and REP-J contexts. Subjects were told that the experiment would consist of four blocks, that stimuli in each block would come one after another with a short silence (~3 s) between stimuli, and that they would be given a break between each ~9 min block. Subjects were instructed to press a button as soon as they detected repetition or a transition in the sound.

Each data collection block contained 4 trials of each of the seven REP conditions, 16 RAND trials, and 4 PINK trials, all randomly interleaved. For each subject, over all four blocks, this amounted to 16 trials of each of the seven REP conditions, 64 RAND trials, and 16 trials of PINK. Importantly, the stimulus for each trial was generated from a different random seed and was therefore unique, with its own target, fillers, and set of jittered intervals. This eliminated the possible confound of longer-term memory effects across multiple trials (Agus et al., 2010). Additionally, in order to reduce the likelihood that any trend found could be explained by the particular noise stimuli that make up a single stimulus set, a different stimulus set was independently generated for each subject.

Stimuli were played through a TDT RM1 mobile processor (Tucker Davis Technologies, Alachue, FL, USA), and presented diotically at 50 dB SPL over Sennheiser HD 650 headphones (Wedemark, Germany). The TDT device delivered the stimuli and recorded button presses, allowing precise reaction times to be measured. Experiments were conducted in a double-walled sound-proof chamber.

Two performance measures were analyzed for all conditions tested: fraction detected and reaction time. The fraction detected is the proportion of trials (out of a total of 16 in each REP or PINK condition) during which the subject pressed the button to indicate detection. Reaction time was measured from the onset of the first target noise to the time of the button press. By dividing reaction time by 0.5 s, one can determine approximately how many noise targets had been presented before detection. “Miss” trials where repetition was present but not detected were excluded from reaction time calculations.

## Results

Twenty-one paid participants aged 20–40 with normal hearing were recruited for this study. Three subjects were authors on this study, and 12 had some musical training. To ensure that subjects were performing the task correctly, subjects with a false alarm rate greater than 50%, calculated as the percentage of all RAND stimuli during which an erroneous detection was reported, were excluded from further analysis, leaving a final total of 17 subjects.

The population false alarm rate, calculated as the proportion of erroneous detections during RAND trials (out of a total of 64) averaged across all 17 subjects, was 17.9%. The average reaction time for PINK trials, was 588 ms.

### Shorter noise targets are harder to detect

**Figure 2:**
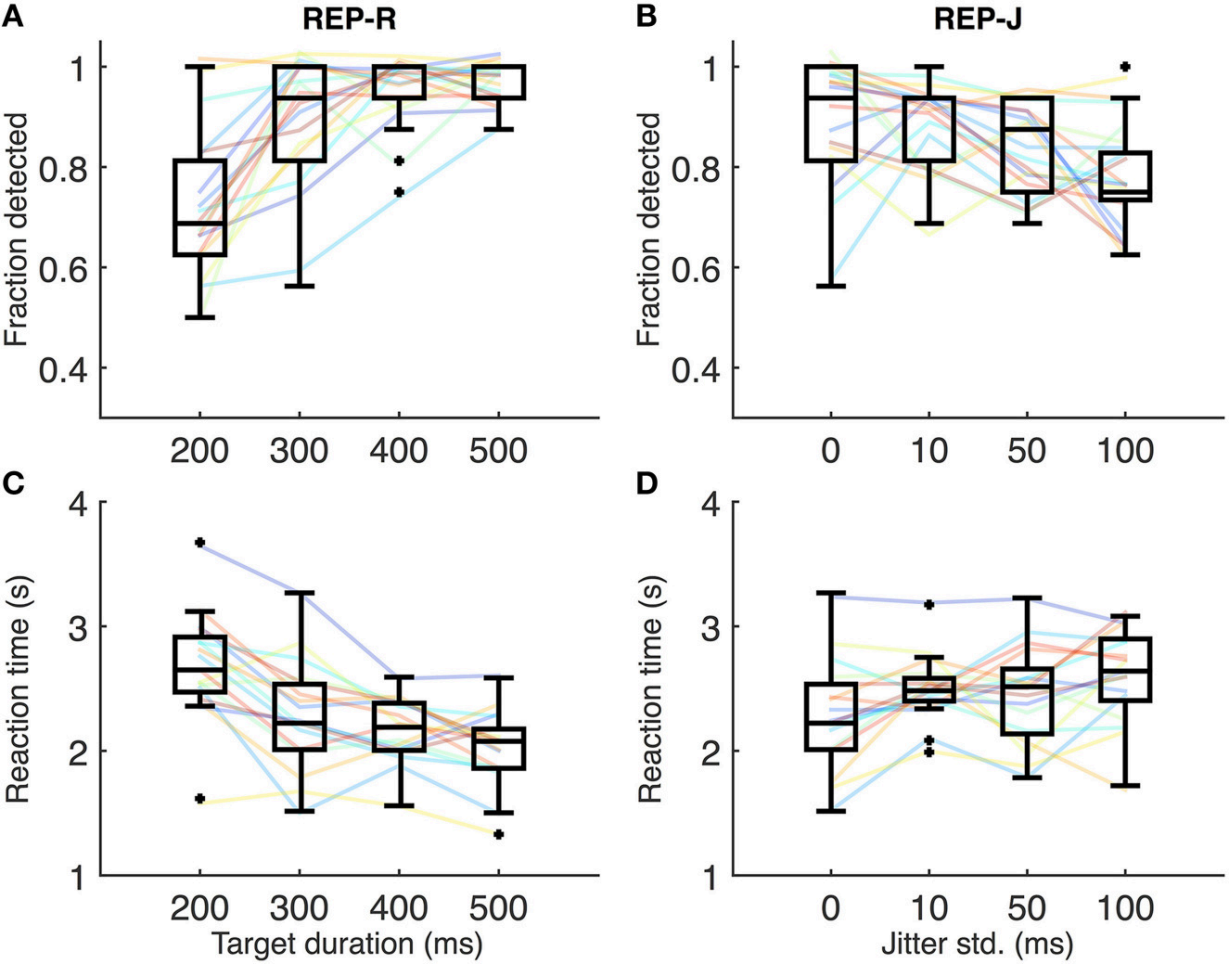
**(A)** Fraction detected for REP-R conditions. Solid lines represent individual subjects, and boxplots summarize the data over the 17 subjects for a given stimulus condition. For display purposes only, a small amount of Gaussian noise (std = 1.5%) has been added to the y-values of the solid lines in **(A–D)** to spread out overlapping data points. **(B)** Fraction detected for REP-J conditions. **(C)** Mean reaction times for REP-R conditions. Solid lines represent mean reaction times for each subject, and boxplots are over the 17 subjects' means. **(D)** Mean reaction times for REP-J.

Figures 2A,C show REP-R detection performance and reaction times, respectively. A few subjects were near 100% detection for all REP-R stimuli, but the overall trend is for detection performance to increase with increasing target duration (Figure 2A), and for reaction time to decrease (Figure 2C). Relative to the shortest target duration (T200-J0: 200 ms target duration, 0 ms jitter), detection performance was significantly higher and reaction times significantly lower (*p* < 0.001 and *p* < 0.01 respectively, *n* = 17 subjects, Wilcoxon signed rank test, Holm-Bonferroni corrected) for all other target durations tested. A significant drop in reaction time was also observed between T300-J0 and T500-J0 (*p* < 0.05, *n* = 17, Wilcoxon signed rank test, Holm-Bonferroni corrected). Thus, as the duration of repeating targets makes up a larger proportion of the IOI, their repetition is more likely to be detected, and fewer target presentations are required for their detection.

### Substantial jitter impairs noise target detection

Figures 2B,D show individual detection rates and reaction times, respectively, for REP-J conditions. Subjects could detect the repeating pattern quite well for all jitter levels, but there are some systematic effects of jitter. We observed no statistically significant differences in reaction time between REP-J conditions (*p* > 0.05, *n* = 17, Wilcoxon signed rank test, Holm-Bonferroni corrected), but detection performance on T300-J100, the most jittered condition, was significantly worse than on T300-J0 and T300-J10 (*p* < 0.05, *n* = 17, Wilcoxon signed rank test, Holm-Bonferroni corrected). Thus, a substantial amount of jitter makes a repeating target more difficult to detect, but modest amounts of jitter appear to be well tolerated.

### What about natural sounds?

We motivated this study of pattern-in-noise detection by considering the ecological need to detect rhythmic natural sounds, such as footsteps, out of background noise. Rhythmic structure is often a hallmark of locomotion or vocal behavior of animate sound sources, and an ability to detect rhythmic patterns may have evolved to facilitate detection of another animal's activity. This could confer a competitive advantage by signaling the presence of potential mates, prey, or predators. Our experimental results indicate that pattern detection benefits from temporal regularity, and it would provide some context for our findings to explore how much temporal jitter is present in natural sounds. We analyzed step interval data from normally walking healthy humans, compiled from three separate studies (Frenkel-Toledo et al., 2005; Yogev et al., 2005; Hausdorff et al., 2007), available on the PhysioNet database (Goldberger et al., 2000). The dataset logged pressure sensor data recorded from underneath both feet, and we defined foot strikes to occur each time pressure under either foot crossed a threshold. Footstep intervals were calculated as the time between successive foot strikes. Since participants were pacing back and forth through a hallway, the need to turn around introduced some footstep intervals that were clear outliers from an otherwise tight distribution. Hence, as an outlier-proof measure of the jitter in step intervals, we calculated the median percentage deviation from the median step interval for each individual. These median deviations ranged from 1.6 to 6.6% across the 72 subjects, with a median of 1.9%. The entire range is less than the median deviation of the intermediate jitter condition (T300-J50) of 6.7%. From this we can conclude that, at least for this class of rhythmic natural sounds, the amount of temporal jitter present would be too small to impair detection performance.

## Discussion

Firstly, we found that for a fixed IOI, a repeating target noise becomes easier to detect as its duration increases. This is consistent with the findings reported in Kaernbach (2004) and may be due to increased signal to noise for longer duration target noises. Secondly, we found that detection performance declines with substantial amounts of temporal jitter (more than the amount of jitter found in footsteps), though pattern detection was remarkably robust to levels of jitter below this level.

### Does repetition detection rely on synaptic memory traces of recent inputs?

Agus et al. (2010) suggested that memory traces that are presumably needed for repetition detection may involve spike-timing dependent plasticity (STDP). Networks incorporating STDP have been shown to quickly learn to detect a repeating pattern of afferent spiking activity amidst otherwise stochastic firing (Masquelier et al., 2008). This makes STDP an appealing candidate mechanism consistent with experimental observations made to date, with two possible caveats. First, subjects were able to recognize repetition with only two presentations of a frozen noise target (Agus et al., 2010), a performance that is so far unmatched by existing models of STDP. Secondly, a purely STDP based model would accurately detect noise targets equally well whether they arrive at regular intervals or not, which is in contrast to our finding that temporal regularity results in better detection performance. This does not rule out that STDP may have a role to play, but it does suggest that it alone does not account for all aspects of noise learning, and indeed Agus and Pressnitzer (2013) suggested the possibility that sensitivity to amplitude modulations may also be involved.

### Modulation filterbanks as an alternative mechanism

A mechanism that would potentially account for the timing aspect of our findings is a modulation filterbank, which is a set of neural filters tuned to different frequencies of modulation of the sound envelope (typically within a frequency band). Modulation filterbank models of the auditory system have shown good agreement with human psychoacoustic data on amplitude modulation detection (Dau et al., 1997) and speech intelligibility (Jørgensen and Dau, 2011), and electrophysiological evidence for modulation tuning exists at various levels of the auditory system (Schreiner and Urbas, 1986, 1988; Kilgard and Merzenich, 1999; Joris et al., 2004). We propose that the brain relies at least in part on modulation filters to detect repetitions in noise, and that the performance decrease we observe in the presence of jitter might be explained by the fact that jittered stimuli will drive modulation filters less strongly. We illustrate the plausibility of this idea through the following analysis.

### Repetition of frozen noise targets results in distinct peaks in the modulation spectrum

**Figure 3:**
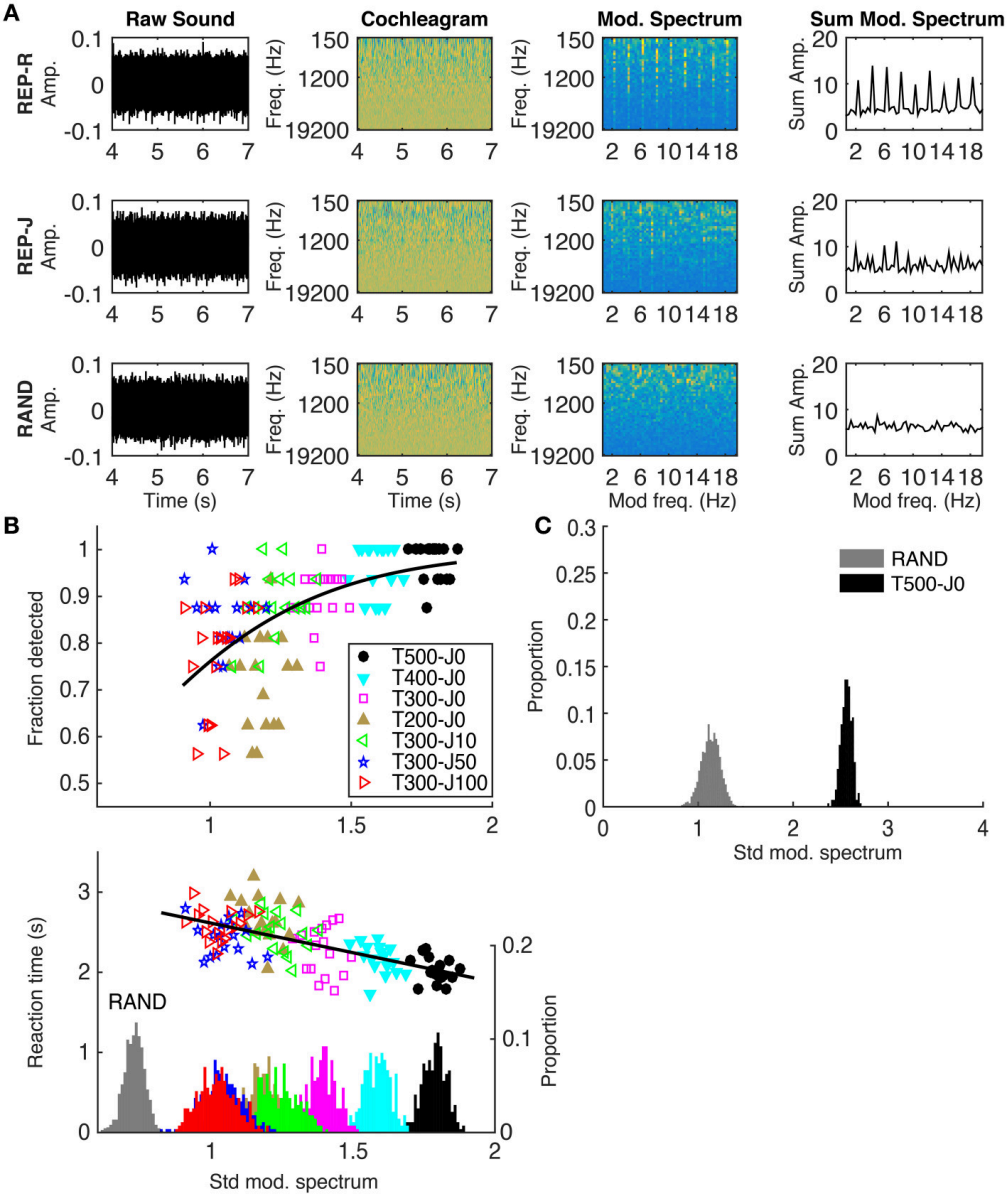
**(A)** Method used to quantify the “peakiness” of the modulation spectrum, shown for one example from REP-R (T300-J0), REP-J (T300-J100), and RAND. From left to right: the raw sound; cochleagram; modulation spectrum in each frequency channel; modulation spectrum summed over frequency channels. Note the presence of vertical stripes at 2 Hz and its harmonics in the modulation spectra of both REP stimuli, and their absence from RAND. The standard deviation of the summed modulation spectrum was calculated for each stimulus trial. **(B)** *Top:* Fraction detected plotted against the standard deviation of the summed modulation spectrum for all trials, conditions, and subjects. Each point represents the fraction detected, out of the nearest-neighboring 16 stimulus examples (regardless of subject) along the x-axis from within the same condition. Different colors represent the different conditions, as shown in the legend. Black line is the fit from a logistic regression. *Bottom:* Reaction time in seconds plotted against the standard deviation of the summed modulation spectrum. For display purposes, the average (clumped) reaction times of each 16 nearest neighbors along the x-axis from the same stimulus condition are plotted. The black line is a linear regression run on all (un-clumped) data. On the secondary y-axis is a histogram showing the distribution of all (un-clumped) standard deviation values for all stimuli within each condition. **(C)** A histogram showing the distribution of standard deviation values calculated over 1 s intervals during RAND (gray) and T500-J0 (black), analogous to the stimuli used in Agus et al. (2010). Note the larger standard deviation values for both conditions in **(C)** using a 1 s window compared to the 3 s window used in **(B)**. For our examples of RAND (*n* = 1088) and T500-J0 (*n* = 272), we see no overlap, suggesting that the modulation spectrum would contain enough information to detect repetition from a single repeat of a 500 ms frozen noise target.

We calculated the modulation spectrum for each stimulus and used the standard deviation of the modulation spectrum as a measure of its “peakiness.” A peaky modulation spectrum would indicate that some modulation filters are being driven more strongly than others, and we sought to investigate whether this correlated with detection performance using the method illustrated in Figure 3A. The first step was to transform each sound stimulus into a simple approximation of the activity pattern received by the auditory pathway by calculating a sound's log-scaled spectrogram (‘cochleagram’). For each sound, the power spectrogram was taken using 20 ms Hanning windows, overlapping by 10 ms. The power across neighboring Fourier frequency components was aggregated using overlapping triangular windows comprising 43 frequency channels with center frequencies ranging from 150 to 19,200 Hz (1/6 octave spacing). Then, the log was taken of the power in each time-frequency bin, and finally any values below a low threshold were set to that threshold. These calculations were performed using code adapted from melbank.m^2^.

The cochleagram was calculated over a 3 s window starting 4 s into the sound, by which time frozen noise targets must have ensued in all REP conditions. The magnitude spectrum of the activity in each frequency channel was calculated and then summed across frequency channels to get the overall modulation spectrum of the sound. We then calculated the standard deviation of the modulation spectrum (≤ 20 Hz) to quantify how much it deviated from a “flat” modulation spectrum. Gaussian white noise without repeating frozen noise targets (our RAND condition) should have a flat modulation spectrum and small standard deviation, while isochronously presented targets (our REP-R conditions) should introduce significant peaks in the modulation spectrum, increasing its standard deviation (Figure 3A, rightmost column). As illustrated in Figure 3B, REP stimuli with longer or more regularly spaced targets had “peakier” modulation spectra and were more reliably and more quickly detected by our subjects. Peakiness of the modulation spectrum correlates significantly with detection (*p* = 0.03, *n* = 7 conditions by 17 subjects = 119, logistic regression), and with faster reaction times (*p* < 10^−20^, *n* = 1649, Pearson correlation). No significant trends were found in fraction detected or in reaction times within individual conditions (*p* > 0.05 in all cases, Pearson correlation).

The analysis in Figure 3B is consistent with the idea that modulation filter type mechanisms could be responsible for the detection of repetition in noise, but it of course does not prove that physiological modulation filters are the only possible mechanism. For example, one might wonder whether autocorrelation models, which are often invoked to describe the processing of periodicities of sounds in the pitch range, might not provide equally good or perhaps even better alternative candidate mechanisms. In digital signal processing, autocorrelations are normally computed by quantifying the similarity of incoming signals to a delayed copy of the input, which is held in memory with complete accuracy for whatever delay period may be required, perhaps up to several seconds. How such highly accurate and flexible auditory short-term memory banks might be implemented using known neurobiological signal processing mechanisms is far from obvious. Nevertheless, we cannot exclude the possibility that the mechanisms that the brain uses to detect recurrent patterns in noise may operate in ways that resemble an autocorrelator more than a modulation filter bank.

### Could a modulation filterbank model account for previous findings?

As mentioned earlier, Kaernbach (2004) and Agus et al. (2010) both demonstrated that human listeners could detect repetition in a 1 s long stimulus where a 500 ms noise token was played only twice. A question worth asking is whether “peakiness” in the modulation spectrum could still be helpful even when there are so few repeats. Figure 3C shows that the standard deviation of the modulation spectrum calculated from 1 s (two period) segments taken from our analogous T500-J0 stimuli do indeed differ substantially from equivalent 1 s segments from non-repeating RAND stimuli, suggesting that peaks in the modulation frequency domain could have provided a useful cue in the aforementioned studies. However, modulation filters alone would not account for the observation in Agus et al. (2010) that noise memory traces can have surprisingly long lasting effects. Thus, both modulation filter-like mechanisms and long term plasticity are likely to be required to fully account for our ability to detect patterns in noise.

Further work is needed to confirm whether modulation filters indeed underlie the results reported here, as well as in other related psychoacoustic studies. For example, timing predictability is also an important cue during auditory scene analysis (Bendixen, 2014), and different patterns of activity across frequency channels in the modulation frequency domain could be involved in the tendency for temporally jitter to cause streams to segregate (Andreou et al., 2011; Rajendran et al., 2013). An additional consideration is the evidence for the oscillatory nature of temporal attention and its effect on task performance, which has been studied both in the visual (Correa et al., 2006; Lakatos et al., 2008) and auditory (Jones et al., 2002; Lakatos, 2005; Jaramillo and Zador, 2011; Henry and Obleser, 2012; Lakatos et al., 2013; Lawrance et al., 2014) domain.

## Conclusions

Our results demonstrate that the ability to detect a repeating pattern is affected by the regularity of timing with which repeated sounds are presented. Specifically, we found that at a presentation rate of 2 Hz, applying a temporal jitter of 20% to the onsets of the noise targets significantly hindered their detection. We also found that the amount of jitter present in natural sounds such as footsteps is likely too small to be detrimental to detection. Finally we showed that aspects of perceptual performance in our study and in other noise pattern detection studies can be well accounted for by the hypothesis that the auditory system uses low frequency modulation filters to detect rhythmic patterns. All together, we conclude that temporal regularity aids in detecting subtle structure in sound.

## Author contributions

VR, NH, KA, JS designed the study, VR, KA acquired the data, VR, NH, JS analyzed the data, interpreted the results, and wrote the manuscript.

### Conflict of interest statement

The authors declare that the research was conducted in the absence of any commercial or financial relationships that could be construed as a potential conflict of interest.

## Footnotes

^1^http://www.auditoryneuroscience.com/patternsinnoise

^2^http://www.ee.ic.ac.uk/hp/staff/dmb/voicebox/voicebox.html
